# Accurate prediction of clinical stroke scales and improved biomarkers of motor impairment from robotic measurements

**DOI:** 10.1371/journal.pone.0245874

**Published:** 2021-01-29

**Authors:** Dimitris K. Agrafiotis, Eric Yang, Gary S. Littman, Geert Byttebier, Laura Dipietro, Allitia DiBernardo, Juan C. Chavez, Avrielle Rykman, Kate McArthur, Karim Hajjar, Kennedy R. Lees, Bruce T. Volpe, Michael Krams, Hermano I. Krebs

**Affiliations:** 1 Janssen Research & Development, Titusville, New Jersey, United States of America; 2 Novartis Institutes for BioMedical Research, Cambridge, Massachusetts, United States of America; 3 GSL Statistical Consulting, Ardmore, Pennsylvania, United States of America; 4 Bioconstat Bvba, Gent, Belgium; 5 Department of Mechanical Engineering, Massachusetts Institute of Technology, Cambridge, Massachusetts, United States of America; 6 Biogen-Idec, Cambridge, Massachusetts, United States of America; 7 Burke Medical Research Institute, White Plains, New York, United States of America; 8 Institute of Cardiovascular and Medical Sciences, University of Glasgow, Glasgow, United Kingdom; 9 Department of Neurology, University of Duisburg-Essen, Essen, Germany; 10 Feinstein Institute for Medical Research, Manhasset, New York, United States of America; South China University of Technology, CHINA

## Abstract

**Objective:**

One of the greatest challenges in clinical trial design is dealing with the subjectivity and variability introduced by human raters when measuring clinical end-points. We hypothesized that robotic measures that capture the kinematics of human movements collected longitudinally in patients after stroke would bear a significant relationship to the ordinal clinical scales and potentially lead to the development of more sensitive motor biomarkers that could improve the efficiency and cost of clinical trials.

**Materials and methods:**

We used clinical scales and a robotic assay to measure arm movement in 208 patients 7, 14, 21, 30 and 90 days after acute ischemic stroke at two separate clinical sites. The robots are low impedance and low friction interactive devices that precisely measure speed, position and force, so that even a hemiparetic patient can generate a complete measurement profile. These profiles were used to develop predictive models of the clinical assessments employing a combination of artificial ant colonies and neural network ensembles.

**Results:**

The resulting models replicated commonly used clinical scales to a cross-validated R^2^ of 0.73, 0.75, 0.63 and 0.60 for the Fugl-Meyer, Motor Power, NIH stroke and modified Rankin scales, respectively. Moreover, when suitably scaled and combined, the robotic measures demonstrated a significant increase in effect size from day 7 to 90 over historical data (1.47 versus 0.67).

**Discussion and conclusion:**

These results suggest that it is possible to derive surrogate biomarkers that can significantly reduce the sample size required to power future stroke clinical trials.

## Introduction

Stroke is the leading cause of permanent disability in the United States [1]. With the demographic profiles of most developed countries shifting towards older individuals, disability due to stroke is projected to increase significantly in future years. As such, there is great interest in finding pharmacological agents to promote neuro-protection and neuro-recovery as well as in reducing the cost of clinical trials [2]. We chose to investigate the use of robotic systems because of their growing adoption in stroke wards to deliver therapy. However, many robotic devices, such as the InMotion Arm [3], afford not only the possibility to promote faster and better rehabilitation, but also the potential to track an individual’s progress [4–6].

An important potential advantage of robotic devices over “traditional” clinical instruments is that the measurement variability due to the skills and expertise of the rater can be removed from the assessment process. It has been shown repeatedly that standard clinical scales, such as the Fugl-Meyer assessment (FM) [7], show a high degree of variability among different raters [8], ultimately leading to larger sample sizes being required to demonstrate the value of a particular intervention [9]. Thus, the ability to remove inter- and intra-rater variability as well as conduct the assessments more efficiently would enable faster and less costly clinical trials [10].

However, the correlation between robotic assays and established clinical scales—such as the NIH stroke scale (NIH) [11], the modified Rankin scale (MR) [12, 13], or the FM—remains an open question. Although these scales have some important shortcomings, their use has been widespread and there are a large number of legacy trials that have recorded one or all of them. To properly leverage the information captured in these historical trials, we need a method for mapping the results from the robotic measurements to these conventional clinical assessments. Furthermore, because robotic assays have not been approved for use as clinical endpoints by the FDA, it must, at a minimum, be demonstrated that they capture information similar to that of the currently approved instruments. Prior work has shown that robotic measures correlated well with the FM [14]. However, those were derived from chronic stroke during a period when most pharmacological interventions ceased. Additionally, that work employed linear combinations of different robotic measurements [14–18]. Although the correlations were good, they suggested the potential to construct a much improved and more accurate predictor using non-linear techniques specifically neural networks. The central goal of this work was to demonstrate that we can use the same metrics to reconstruct existing clinical scales as well as derive composites that are more sensitive the change over time as patients recover from stroke. Given the limitations of existing stroke scales [19, 20], we believe that with the advent of new digital biomarkers, we can do better.

Here, we provide methods and modeling details of a longitudinal study involving 208 patients who had suffered severe to moderate acute ischemic stroke and were assessed with four commonly used clinical instruments [21]–NIH stroke scale (NIH), Fugl-Meyer assessment (FM), Motor Power (MP) [22, 23] and modified Rankin scale (MR)–as well as with a robotic assay to measure arm movement 7, 14, 21, 30, and 90 days after the stroke onset. We hypothesized that by utilizing nonlinear models obtained through a rigorous feature selection algorithm, we could improve our prediction of the standard clinical scales. We hypothesized that the different clinical scales are in part functions of gross motor movements that are nonlinear combinations of individual movement components that are being recorded by the robotic apparatus [24]. Thus, for any effective reconstruction of these clinical scales, a nonlinear modeling strategy must be adopted. Here, we utilize artificial ant colonies and neural network ensembles to reconstruct the clinical scores with a high degree of accuracy that is comparable to the level of agreement among different expert raters (cross-validated R^2^ of 0.75, 0.73, 0.63 and 0.60 for MP, FM, NIH and MR, respectively). Further, we demonstrate that the robotic measurements, due to the use of systematic and objective measurements, are able to reduce variability even in the case of a single highly trained rater.

## Methods

### Study population

In this study, 208 patients who had suffered acute stroke (defined as patients with a baseline NIH of 7–20 recorded at day 7 days since stroke onset) were enrolled and were given a battery of standard clinical assessments including the NIH, FM, MR and MP [25] measured only on the affected side as per standard practice. Of these four, two clinical scales are of prime interest to us, the NIH and the FM. These patients were evaluated on days 7, 14, 21, 30, and 90 after the initial stroke. The subjects were distributed between two sites: the Burke Rehabilitation Center in New York (145 patients) and the Western Infirmary in Glasgow, Scotland (63 patients). To minimize inter-rater variability, the patients were assessed by a single highly trained clinician at Burke and two highly trained clinicians at Glasgow. In addition to the clinical scales, each of the patients was also evaluated with the InMotion robot [3], a commercial version of the MIT-Manus robot, to obtain a battery of robot-measured kinematic and kinetic (RMK) variables. For the RMK battery, each patient was evaluated twice, once on the side of the body affected by the stroke and once on the unaffected side, in order to explore any potential relationships between them. The RMK measurements for each patient took approximately 60 minutes to complete (40 minutes for the affected side, which for severe patients required assistance with each move, and 20 minutes on the unaffected side), whereas the standard clinical assessments took as long as 90 minutes.

### Robot measured kinematics and kinetics

The RMK battery consists of several metrics derived from various directed unassisted reaching tasks, circle drawing, resistance to external forces, and shoulder strength measurement. These metrics are listed in Table 1. The directed reaching tasks are broken down into 8 macro-metrics and 6 micro-metrics [26]. The macro-metrics involve metrics such as the deviation from a straight line when reaching for different targets (“Deviation”), aim to the targets (“Aim”), movement average (Mean Speed), peak speed (“Peak Speed”) and duration (“Duration”) of movement, and three different smoothness metrics which involve the movement mean speed divided by the peak speed (“Smooth M/P”), a jerk metric which corresponds to the magnitude of jerk divided by the peak speed (“Smooth J1”–best for discrete movements), and a jerk metric which corresponds to the root mean square of the jerk normalized by the duration of the movement (“Smooth J2”–best for rhythmic movements). It has been demonstrated that unconstrained discrete and rhythmic reaching movements of healthy subjects minimize jerk [27, 28].

**Table 1:** Overall description of the different RMK metrics.

Measurement	Metrics	Abbreviation	Additional Description
Primary Motion	Aim	Aim	
	Deviation of Path	Deviation	Maximum distance between straight-line path vs. patient motion
	Average Speed	Mean Speed	
	Peak Speed	Peak Speed	
	Movement Duration	Duration	Time To Reach Target
	Jerk Metric	Smooth M/P	Mean Speed/Peak Speed
	Jerk Metric 1	Smooth J1	Jerk Metric Normalized by Peak Speed
	Jerk Metric 2	Smooth J2	Jerk Metric Normalized by Duration
	Circle Drawing	Ellipse	Difference between major and minor axis for a drawn circle
Sub-Movements	Number of Sub-movements	Numb Subm	Number of sub-movements
	Duration of Sub-movements	Dur Subm	Average Width of sub-movement velocity profile
	Sub Movement Overlap	Overlap Subm	Degree of Overlap between sub-movements
	Sub Movement Peak	Max Subm	Maximum Height of the sub-movements
	Sub Movement Skewness	Sigma Subm	Statistical Skewness of sub-movements
	Sub Movement Intervals	Dist Subm	Interpeak Interval of sub-movements
Power	Static Resistance	Plbck	Resistance against force generated by robot
	Dynamic Resistance	Rnd Dyn	Average distance moved vs. Set Resistance Level
	Shoulder Strength	Mean Z	Resistance against force generated by robot in the vertical direction

The same reaching tasks are broken down further into sub-movements as described by Novak et al [29]. There are then decomposed into various micro-metrics such as the number of sub-movements (“Numb Subm”), duration of sub-movement (“Dur Subm”), degree of sub-movement overlap (“Overlap Subm”), sub-movement peak (“Max Subm”), inter-peak interval (“Dist Subm”), and sub-movement skewness (“Sigma Subm”). The circle drawing task yields a metric relating the ability to coordinate the shoulder and elbow independently, characterized by the ratio of the major to the minor axis of an ellipse fitted to the attempted circle drawing (“Ellipse”). Finally, the movement against resistance evaluates the ability of the subject to move the actuator against a particular level of robotic resistance (“Rnd Dyn Mean Dist Measure”) as well as the ability to keep the robotic actuator still while the robot attempts to move the actuator (“Plbck Mean”). This leads to a total of 17 individual metrics being tracked by the RMK system. Given that the RMK metrics are evaluated for both the affected and unaffected sides, this leads to a total of 34 robotic variables that are captured for each patient. This set is completed by one final kinetic variable called Mean Z (“Mean Z”), which measures the mean shoulder strength (Z force) for flexion, extension, abduction, and adduction. Because of the sensor’s range and the fact that most patients would hit the instrument ceiling on their unaffected arm, Mean Z was measured only on the affected side.

Since the RMK endpoints used a variety of units and the assessments fell across widely divergent ranges, each endpoint was linearly normalized from 0 to 1 using the formula:

$$x^{\prime }(r,p,t)=(x(r,p,t)-\;\underset{p,t}{\text{min}}x(r,p,t))/(\underset{p,t}{\text{max}}x(r,p,t)-\underset{p,t}{\text{min}}x(r,p,t))$$ (1)

where *x*(*r*, *p*, *t*) represents the measurement of the *r*-th RMK variable for the *p*-th patient at the *t*-th time point, and min(.) and max(.) run across all patients and time points for that variable.

### Feature selection

As mentioned earlier, our working assumption is that the different clinical scales are functions of gross motor movements which are implicitly captured by the various RMK variables recorded by the robotic apparatus. To test this hypothesis, we used a machine learning approach aimed at predicting the clinical scores of a given patient on a given day from the RMK variables measured for that patient on that same day. Models were derived independently for each clinical scale, as different scales may capture different aspects of motor movement and thus require a different subset of RMK variables for effective reconstruction. Each patient contributed at most 6 records to the training set, one for each day for which an RMK and clinical assessment were made (days 7, 14, 21, 30 and 90 plus some patients were also evaluated at day 3). If either the clinical score or any of the RMK variables were missing, that record was excluded from the data set. (The day on which the measurement was made was not included as an independent variable.).

To build robust models, one must guard against over-fitting. Over-fitting arises when the number of features or adjustable parameters in the model substantially exceeds the number of training samples. The presence of excessive features can cause the learning algorithm to focus attention on the idiosyncrasies of the individual samples and lose sight of the broad picture that is essential for generalization beyond the training set. A common solution to this problem is to employ a feature selection algorithm to identify a subset of relevant features and use only them to construct the actual model [30]. An exhaustive search is usually impractical since it involves 2^*N*^ possible combinations where *N* is the total number of available features, or $\frac{N!}{(N-K)!K!}$ combinations if the desired number of features *K* is prescribed. Several feature selection algorithms have been devised ranging from simple greedy approaches, such as forward selection or backward elimination, to more elaborate methodologies, such as simulated annealing, evolutionary programming and genetic algorithms [31].

In the present work, we use a feature selection algorithm based on artificial ant colonies that was originally designed to model the biological properties of chemical compounds [32, 33]. Algorithms based on artificial ant systems are inspired by the fact that real ants, using deposits of pheromone as a communication agent, are able to find the shortest path between a food source and their nest [34]. A moving ant marks its path by depositing pheromone on the ground. Although each individual ant moves at random, it can detect pheromone trails and follow one of them with a probability proportional to the amount of pheromone on the trail. By adding its own pheromone deposits, the ant reinforces the trail and makes it more attractive to the other ants. While all paths are initially equally probable, the shorter ones encounter more ants making round trips to the food source per time unit and, therefore, receive more pheromone. Thus, short paths become increasingly more attractive to the ants, and eventually all ants follow the shortest trail.

For feature selection, we consider the selection of a variable as a step of the real ant’s path; therefore, the whole path represents a choice of a particular subset of *K* variables out of all *N* variables. Each variable *k*, *k = 1…N*, is assigned a weight *w*_*k*_ that is used to calculate the probability *p*_*k*_ with which the variable is randomly selected by an ant. Initially, the weights (and probabilities) for all variables are equal, (*w*_*k*_ *= w*_*0*_ for all *k*). After the first ant has selected a subset of *K* variables, a model is built using those variables and the quality of that model is used to compute the “length” *L* of the ant’s path (the better the quality of the model, the shorter the path).

**Fig 1:**
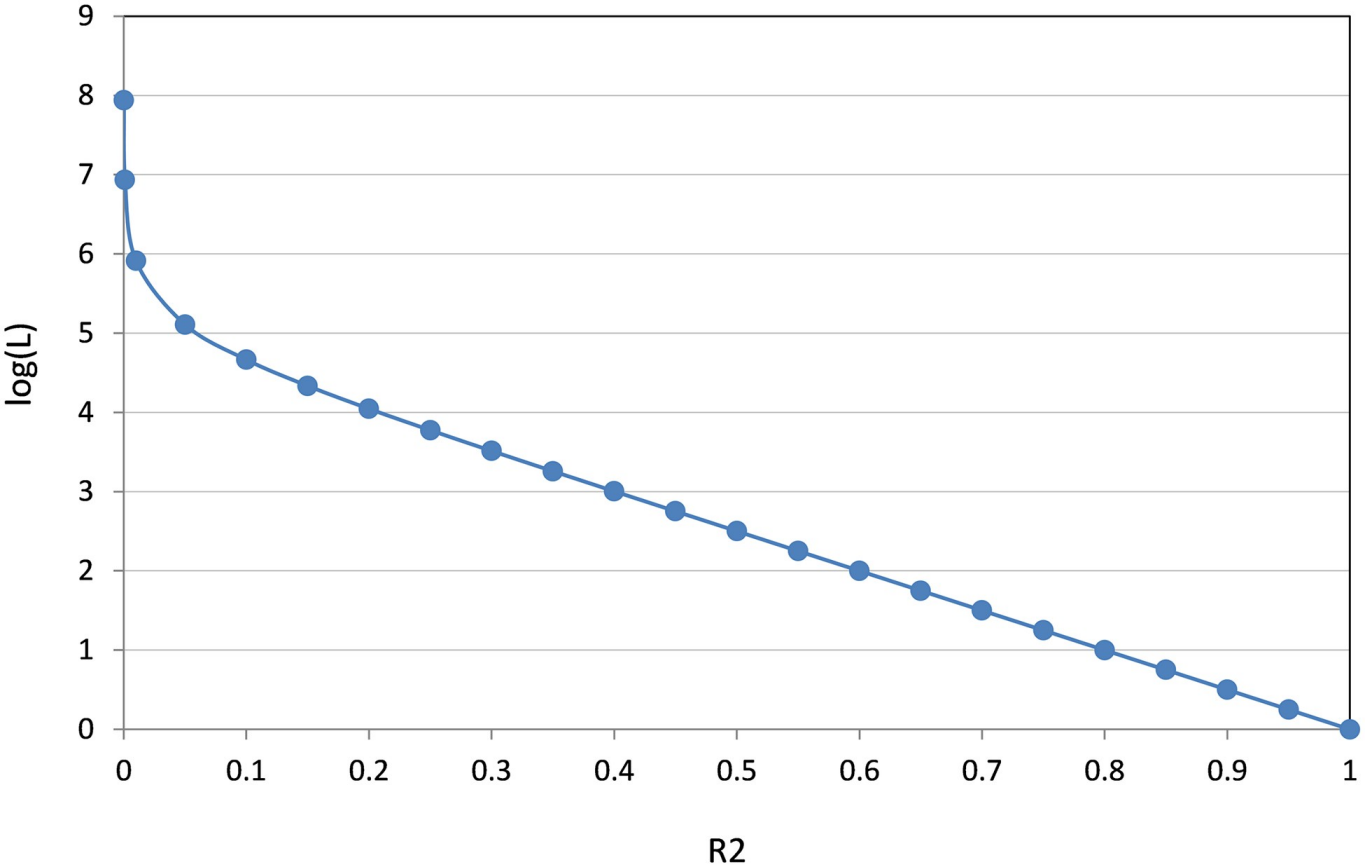
Dependence of the length of the ant’s path L (log scale) on the value of R^2^. As can be seen from this plot, L increases 10-fold as R^2^ decreases by 0.2 units up to a R^2^ value of ~0.2, and at a much greater rate for R^2^ values lower than 0.2.

The path length *L* is derived from the model’s training *R*^2^ using Eq 2:

$$L(R^{2})=\frac{10^{5}-1}{10^{5R^{2}}-1}\;$$ (2)

which increases 10-fold as *R*^2^ decreases by 0.2 up to a *R*^2^ value of ~0.2, and thus provides adequate differentiation between feature subsets, while more severely penalizing solutions with *R*^2^ values lower than 0.2 (Fig 1). This formula was derived empirically and has worked well in other similar contexts [30, 31].

After *L* has been calculated, the weights corresponding to the selected variables are updated according to the following rule:

$$w_{k}(t+1)=(1-\rho )w_{k}(t)+\frac{\Delta w}{L}\;$$ (3)

where *t* is the ant’s number, *ρ* is the evaporation coefficient that simulates the evaporation of the pheromone from the real ants’ paths, and Δ*w* is a constant factor. The next ant calculates the probabilities *p*_*k*_ using the updated weights according to Eq 3:

$$p_{k}=\frac{w_{k}}{\sum_{k}w_{k}}$$ (4)

The process is repeated for the specified number of ants, and the best selection found is reported. Variables that contribute to good solutions (small *L*, high R^2^) end up with larger weights. Thus, these variables tend to be selected more often, and the overall quality of solutions increases as the simulation progresses.

In this work, we used 3,000 ants, and set the initial weights *w*_*k*_(0) to 0.01 and the weight increment Δ*w* to 0.1. Because of its stochastic nature, this process was repeated 10 times to minimize the likelihood of accidental convergence to a poor local minimum. The features identified by each run were used in a subsequent step to construct an ensemble of 10 neural networks, and each ensemble was cross-validated 10 times (see below).

### Neural networks

For each candidate set of *K* features, a model was derived using three-layer, fully connected feed-forward artificial neural networks comprised of *K* inputs, one output, and one hidden layer, and trained using the standard error back-propagation algorithm [35]. The logistic transfer function *f*(*x*) = 1/(1 + *e*^−*x*^) was used for both hidden and output layers. Each network was trained for 100 epochs, using a linearly decreasing learning rate from 1.0 to 0.01 and a momentum of 0.8. During each epoch, the training patterns were presented to the network in a randomized order.

Neural networks were chosen because of their ability to capture complex nonlinear relationships. However, neural networks are inherently unstable in that small changes in the training set and/or training parameters can lead to large changes in their generalization performance. A proven way to improve the accuracy of unstable predictors is to create multiple instances of them and aggregate their predictions [35]. So-called ensemble techniques, such as bagging [36], boosting [37], and stacking [38, 39], combine multiple models to achieve better predictive performance than could be obtained from any of the constituent models. Obviously, combining the output of multiple predictors is useful only if there is disagreement between them. Model diversity can be introduced by combining different learning algorithms, varying the input features, randomizing the training procedure, adding noise to the response value, or manipulating the training set.

**Fig 2:**
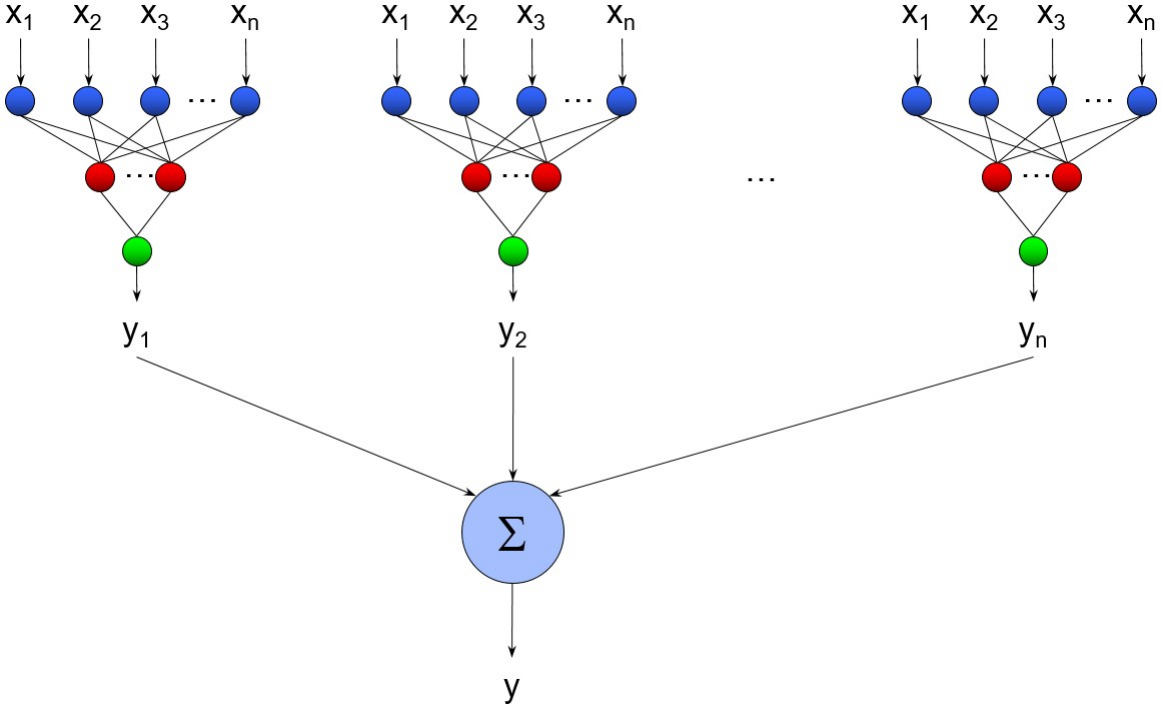
Schematic illustration of the ensemble neural network models used to predict the clinical scales from the RMK variables. Each subset of features identified by the artificial ant algorithm was used to construct 10 independent neural network models using exactly the same network topology and training parameters but a different random seed number (and thus different initial synaptic parameters and presentation sequence of the training samples). The predictions of these 10 models were averaged to produce the aggregate prediction of the ensemble.

In the present work, each subset of features identified by the artificial ant algorithm was used to construct 10 independent neural network models using exactly the same network topology and training parameters but a different random seed number (and thus different initial synaptic parameters and presentation sequence of the training samples). The predictions of these 10 models were averaged to produce the aggregate prediction of the ensemble, as illustrated in Fig 2. We have found that this approach often outperforms alternative ensemble methods [40]. We employed this approach for every single run of the feature selection algorithm.

### Cross-validation

Following common practice, the quality of the models was assessed using 10-fold (leave-10%-out) cross-validation, and quantified using the cross-validated correlation coefficient, *R*^*2*^_*CV*_, which measures the correlation between the actual and the predicted clinical scores. *R*^*2*^_*CV*_ was obtained using the jackknife approach, i.e., by dividing the training data into 10 disjoint subsets each containing 10% of the patterns, systematically removing each subset from the training set, building a model with the remaining patterns, and predicting the clinical scores of the removed patterns using the optimized network parameters. Once all 10 subsets were processed in this manner, the resulting predictions were compared to the original clinical scores to determine their degree of agreement (*R*^*2*^_*CV*_). Since cross-validation is itself susceptible to the particular partitioning of the data samples, each model was cross-validated independently 10 times using a different random shuffling of the training patterns in order to establish a better estimate of its generalization ability. The same cross-validation procedure was used for both individual networks and network ensembles.

### Computational details

Feature selection, neural network modeling and cross-validation were implemented in the C++ and C# programming languages and are part of the DirectedDiversity^®^ [41] and Third Dimension Explorer [42] software suites. Linear models were built in the R statistical programming environment [43].

### Ethics statement

The specific study from which the data has been collected has been expressly approved by the MIT Committee on the Use of Humans as Experimental Subjects (COUHES), the Burke Rehabilitation Hospital IRB, and the NHS National Patient Safety Agency / Gardiner Institute Western Infirmary of Glasgow University IRB. All participants provided written consent to participate in this study, and copies of their signed consent forms have been archived. This consent procedure was approved by all the aforementioned ethics committees/IRBs.

## Results and discussion

### Descriptive statistics and normalization

Our trial had two primary goals: 1) test whether the RMK metrics can predict the clinical scales with sufficient accuracy to serve as their surrogates for measuring impairment and recovery in a non-variable and objective manner, and 2) test whether it is possible to design a more sensitive RMK-based endpoint to measure effect size and thus reduce the sample size of future clinical trials. Endpoint sensitivity was assessed using the standardized paired effect size, defined as the mean divided by the standard deviation of the day 7 to day 90 changes, aggregated over all patients.

To enable these analyses, we identified two complementary patient populations: 1) those with complete data (i.e., no missing values) for days 7 and 90 for all 35 RMK variables and all four clinical scales (87 patients, 67 from Burke and 20 from Glasgow, hereafter referred to as *completers*); and 2) those who did not meet these criteria (121 patients, 79 from Burke and 42 from Glasgow, referred to as *non-completers*). Descriptive statistics of the two subpopulations are provided in Table 2. All four clinical scales show a statistically significant difference in the distribution of the completer and non-completer populations when aggregated over the entire course of the trial, with the former showing a greater degree of impairment. By contrast, no statistically significant difference was observed in the NIH scores upon admission or the FM, MP and NIH scores at baseline (day 7), though the completers appear slightly more impaired on average. In general, different variables showed different directional movement and rates of progression over time, with substantial variability from patient to patient.

**Table 2:** Descriptive statistics.

		Completers						Non-Completers						Total	
		Count	Min	Max	Mean	StdDev	Median	Count	Min	Max	Mean	StdDev	Median	Count	p-value
Demographics	Age	87	29	96	70.552	13.860	73	121	22	97	73.521	13.522	76	208	1.3E-01
	Sex | Male	43						63						106	
	Sex | Female	44						58						102	
	Ethnicity | Caucasian	66						98						164	
	Ethnicity | Hispanic	6						8						14	
	Ethnicity | Asian	1						2						3	
	Ethnicity | African American	14						13						27	
	Handedness | Right	58						80						138	
	Handedness | Left	8						10						18	
	Handedness | Left / Right Writing	0						1						1	
	Handedness | Unknown	21						30						51	
	Affected Side | Right Body	32						40						72	
	Affected Side | Left Body	35						51						86	
	Affected Side | Unknown	20						30						50	
	Site | Burke	67						79						146	
	Site | Glasgow	20						42						62	
Clinical Scales	NIH Admission	51	1	27	10.860	6.470	11	77	1	27	10.390	5.910	9	128	6.8E-01
	FM Day 7	87	4	66	40.250	21.650	45	91	4	66	38.490	21.930	41	178	5.9E-01
	MP Day 7	87	2	70	45.490	20.140	50	73	0	70	39.290	21.710	43	160	6.5E-02
	NIH Day 7	87	0	24	5.750	4.270	5	93	0	21	6.490	4.860	6	180	2.8E-01
	FM	403	4	66	48.790	20.030	58	391	0	66	44.430	22.600	54	794	4.2E-03
	MP	402	2	70	52.270	17.830	56	314	0	70	45.360	21.500	53	716	5.2E-06
	NIH	404	0	24	3.540	3.720	2	408	0	21	4.590	4.500	3	812	3.1E-04
	MR	165	0	5	2.350	1.280	2	129	0	5	2.820	1.250	3	294	1.7E-03
RMK Metrics	Aim Aff	404	0.003	1	0.176	0.122	0.144	377	0	0.79	0.210	0.144	0.172	781	4.1E-04
	Aim NonAff	404	0	0.931	0.245	0.138	0.215	380	0.031	1	0.272	0.151	0.245	784	9.3E-03
	Deviation Aff	402	0.005	0.848	0.093	0.106	0.06	381	0	1	0.128	0.164	0.072	783	4.5E-04
	Deviation NonAff	404	0	1	0.092	0.101	0.067	377	0.001	0.873	0.122	0.141	0.078	781	7.2E-04
	Dist Subm Aff	404	0.01	1	0.364	0.126	0.358	368	0	0.754	0.374	0.135	0.369	772	2.9E-01
	Dist Subm NonAff	404	0.036	1	0.344	0.129	0.334	381	0	0.749	0.376	0.137	0.369	785	8.0E-04
	Dur Subm Aff	404	0.173	1	0.493	0.134	0.491	369	0	0.831	0.479	0.140	0.486	773	1.6E-01
	Dur Subm NonAff	404	0.262	1	0.600	0.134	0.589	381	0	0.977	0.612	0.130	0.613	785	2.0E-01
	Duration Aff	404	0.044	1	0.234	0.134	0.201	380	0	0.894	0.266	0.162	0.226	784	2.8E-03
	Duration NonAff	404	0.035	0.855	0.207	0.126	0.175	381	0	1	0.257	0.149	0.227	785	5.2E-07
	Ellipse Aff	403	0.999	1	1.000	0.000	1	379	0	1	0.992	0.089	1	782	8.1E-02
	Ellipse NonAff	404	0.002	1	0.755	0.183	0.818	380	0	0.988	0.734	0.188	0.793	784	1.1E-01
	Max Subm Aff	404	0.031	0.77	0.339	0.133	0.323	369	0	1	0.319	0.156	0.292	773	5.7E-02
	Max Subm NonAff	404	0.018	0.775	0.321	0.152	0.303	381	0	1	0.283	0.160	0.258	785	6.9E-04
	Mean Speed Aff	404	0.007	0.689	0.299	0.111	0.29	380	0	1	0.281	0.138	0.267	784	4.5E-02
	Mean Speed NonAff	404	0.008	1	0.318	0.136	0.313	377	0	0.814	0.279	0.146	0.269	781	1.3E-04
	Mean Z Aff	404	0.816	1	0.849	0.029	0.842	328	0	0.914	0.829	0.094	0.835	732	2.2E-04
	Numb Subm Aff	404	0	0.858	0.181	0.128	0.156	369	0.008	1	0.218	0.156	0.185	773	3.6E-04
	Numb Subm NonAff	404	0	1	0.161	0.133	0.124	381	0.006	0.922	0.213	0.152	0.179	785	4.5E-07
	Overlap Subm Aff	404	0.056	1	0.453	0.128	0.443	367	0	0.761	0.432	0.125	0.434	771	2.2E-02
	Overlap Subm NonAff	404	0.16	1	0.491	0.149	0.482	379	0	0.93	0.482	0.130	0.48	783	3.7E-01
	Peak Speed Aff	404	0.05	0.833	0.397	0.133	0.377	380	0	1	0.379	0.152	0.36	784	7.9E-02
	Peak Speed NonAff	404	0.057	0.801	0.359	0.150	0.343	377	0	1	0.318	0.155	0.306	781	1.9E-04
	Plbck Mean Aff	404	0	0.961	0.170	0.171	0.098	377	0.003	1	0.212	0.188	0.147	781	1.2E-03
	Plbck Mean NonAff	403	0.003	0.823	0.139	0.170	0.063	383	0	1	0.164	0.167	0.089	786	3.8E-02
	Rnd Dyn Mean Dist Aff	404	0.017	0.975	0.728	0.258	0.866	380	0	1	0.670	0.300	0.86	784	3.9E-03
	Rnd Dyn Mean Dist NonAff	404	0	0.982	0.770	0.120	0.797	381	0.106	1	0.752	0.148	0.796	785	6.3E-02
	Sigma Subm Aff	404	0.165	1	0.456	0.124	0.46	369	0	0.801	0.436	0.124	0.447	773	2.5E-02
	Sigma Subm NonAff	404	0.212	1	0.553	0.132	0.546	381	0	0.905	0.550	0.117	0.55	785	7.4E-01
	Smooth J1 Aff	404	0.029	0.942	0.170	0.101	0.143	375	0	1	0.192	0.128	0.154	779	8.2E-03
	Smooth J1 NonAff	404	0	0.466	0.123	0.059	0.114	381	0.005	1	0.128	0.091	0.109	785	3.6E-01
	Smooth J2 Aff	404	0	0.588	0.114	0.075	0.096	378	0.003	1	0.121	0.118	0.086	782	3.3E-01
	Smooth J2 NonAff	404	0	0.39	0.084	0.055	0.074	380	0	1	0.076	0.077	0.059	784	9.6E-02
	Smooth M/P Aff	404	0	0.967	0.579	0.129	0.601	380	0.111	1	0.550	0.150	0.562	784	3.9E-03
	Smooth M/P NonAff	404	0	1	0.500	0.135	0.524	377	0.017	0.848	0.470	0.147	0.484	781	3.1E-03

RMK metrics are normalized across all patients and assessment points. Statistics for clinical scales and RMK metrics are based on total number of patient assessments. p-values that indicate a statistically significant difference between completers and non-completers are highlighted in red.

We were well aware that in a relatively large group of patients 7 days post stroke the FM might include an occasional patient who showed a ceiling effect for the measurement [20], especially as the accession conditions did not include a damage severity assessment or a minimum motor impairment score. The fact that 5 out of 208 patients had an FM score of 66 by day 7 strengthens the point of this study because the goal was to establish a biomarker that would cover the full spectrum of stroke patients and we were concerned that a study conducted exclusively in the US would be limited to patients with very severe to moderate stroke. The US hospital used in this study is an independent rehabilitation facility admitting referrals from acute hospitals with severe to moderate strokes, whereas the UK hospital is an integrated facility that cares for patients with both acute and chronic stroke. This is reflected in our results which show that the highest FM patient score admitted in our study in the US was 46 (max 66) at day 7, while the mean score of patients admitted to the UK facility was 39.

### Correlation analysis

**Fig 3:**
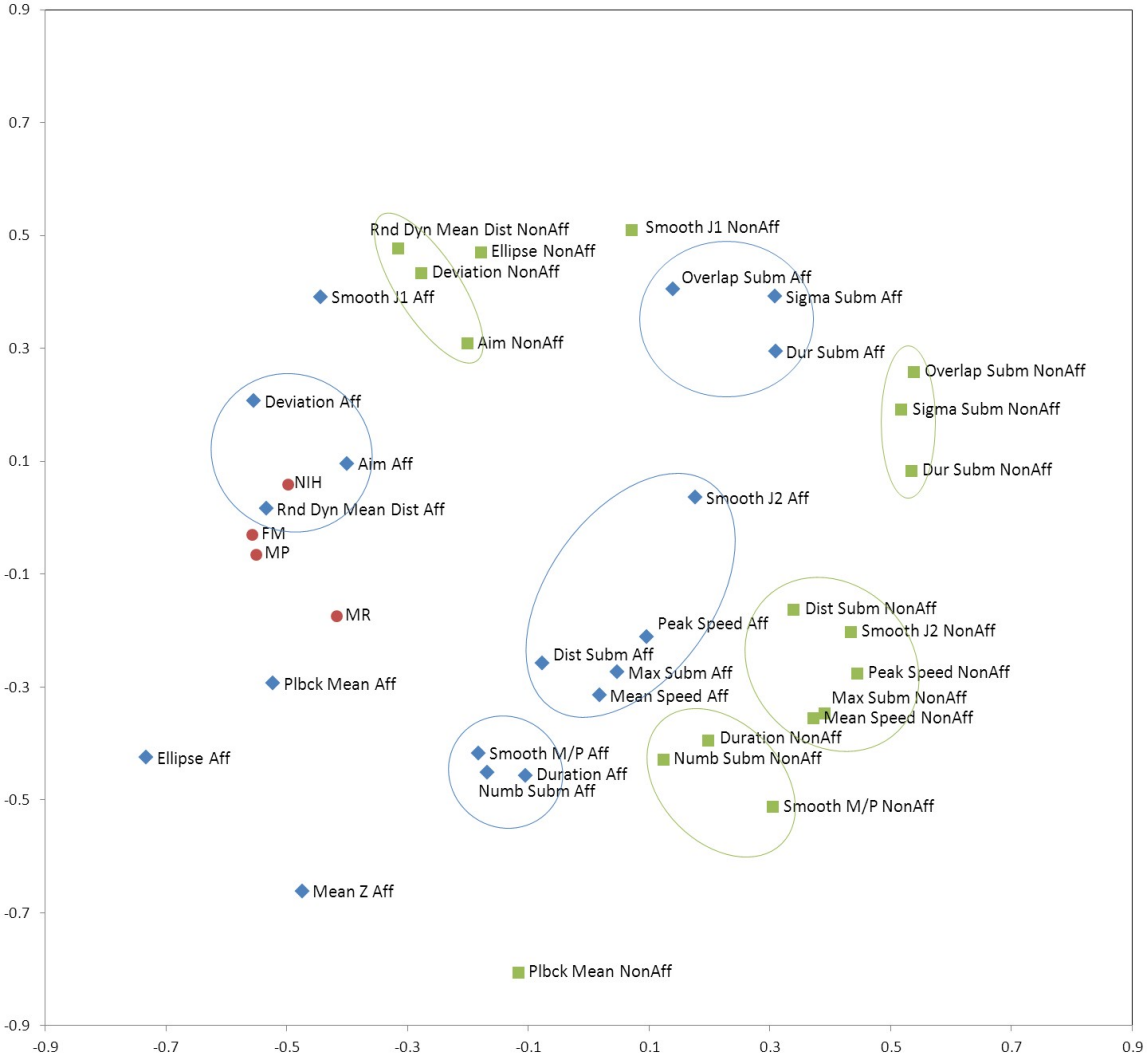
SPE map of the correlation distances of the clinical and RMK parameters for the completers cohort. The map was derived by computing the pairwise Pearson correlation coefficients (R) for all pairs of features, converting them to correlation distances (1-abs(R)), and embedding the resulting matrix into 2 dimensions in such a way that the distances of the points on the map approximate as closely as possible the correlation distances of the respective features. The clinical parameters are highlighted in red, the RMK parameters on the affected side in blue, and the RMK parameters on the unaffected side in green. The map also shows distinct clusters of correlated variables which are preserved on both the affected and unaffected sides (outlined by green and blue ellipses, respectively).

An intuitive way to visualize the correlation structure of the RMK data set is to embed the 35 robotic and four clinical variables into a two-dimensional nonlinear map in a way that preserves as much as possible the pairwise correlations between them. The map shown in Fig 3 contains a point for each RMK and clinical metric and was constructed using stochastic proximity embedding (SPE) [44–46] so that the distances of the points on the map match as closely as possible the correlation distances of the corresponding features, defined as *d*_*ij*_ = 1 –abs(*R*_*ij*_), where *R*_*ij*_ is the Pearson correlation coefficient between the *i*-th and *j*-th features computed over all patients and time points. Thus, correlated (and anti-correlated) features appear close to each other on the map, whereas uncorrelated ones appear further apart (note that the actual distance between two points is a function of not only the correlation between these two features alone, but to all the other features as well–the map reproduces all pairwise distances in a least-squares sense).

Several observations emerge from this map. First, the four clinical scales (highlighted in red) show a substantial degree of correlation to each other as compared to the majority of the RMK variables, with FM and MP exhibiting very similar correlation profiles and being highly correlated themselves (R = 0.933). This is consistent with the findings of Bosecker et al [14]. on chronic stroke patients (111 patients, R = 0.785) as well as previous studies of subacute stroke by Ferraro et al. [47] (12 patients, R = 0.981) and Krebs et al. [8] (56 patients, R = 0.981), and shows the tight coupling of strength (MP) and isolated joint movement (FM).

Second, the RMK variables on the affected side (in blue) exhibit substantially greater correlation to the clinical scales compared to the non-affected side (in green). Among all the RMK metrics, *Rnd Dyn Mean Dist*, *Aim*, *Deviation*, *Smooth J1*, *Plbck Mean*, and *Ellipse* exhibit the highest correlation to the clinical scores both on the affected and the unaffected sides, but substantially more so on the affected side. Finally, the map reveals distinct clusters of correlated variables which are preserved on both the affected and unaffected sides (outlined by green and blue ellipses, respectively). These clusters include: 1) *Overlap Subm*, *Sigma Subm* and *Dur Subm*; 2) *Rnd Dyn Mean*, *Aim*, and *Deviation*; 3) *Peak Speed*, *Mean Speed*, *Max Subm*, *Dist Subm*, and *Smooth J2*; and 4) *Numb Subm*, *Duration*, and *Smooth M/P*. While we only measured FM and MP on the ipsilesional side, these results seem to suggest that motor effects cascade to the contralesional side as well.

Given the degree of redundancy among the RMK metrics, we used principal component analysis (PCA) to estimate the number of underlying independent variables and thus the intrinsic dimensionality of the RMK data. The first 3 PCs account for 59% of the total variance in the data, while 10, 14 and 22 PCs are required to reach the 90%, 95% and 99% levels, respectively. (Note that the limited size of our data set precluded the use of more elaborate geodesic approaches for detecting nonlinear manifolds, such as isometric SPE [45], isomap [48], or locally linear embedding [49]. In general, PCA tends to overestimate the true dimensionality of a data sample.).

### Prediction of clinical scales

Models were derived independently for each clinical scale, using the completer population for training and cross-validation, and the non-completer population for external validation. Since the number of optimal features is not known *a priori* and the generalization error is largely determined by the ratio of training samples to adjustable parameters in the model, feature selection was run with varying numbers of input features *k* (2, 4, 6, 8, 10, 12, 14) and hidden units *h* (1, 2, 3). The artificial ant algorithm was run five times for each combination of *k* and *h*, and the features identified in each run were, in turn, used to derive ensemble models comprising 10 individual predictors. Finally, each resulting ensemble (along with its constituent individual networks) was cross-validated 10 times using 10-fold cross-validation. This entire process was repeated separately for each of the four clinical scales. Thus, our modeling effort involved the generation and training of 4 × 7 × 3 × 5 × 10 × (10+1) × 10 = 462,000 individual neural networks (where 10+1 accounts for the 1 training and 10 cross-validation runs for each network).

The models with two hidden units were slightly better than those with one and virtually identical to those with three, so the remaining discussion is based on the models with two hidden units. Similarly, other training parameters, such as momentum, initial synaptic weights and number of training epochs, had minimal impact on the generalization error and were set to the values outlined in the Methods section. Although, as we discuss later, there were distinct differences among clinical scales, all models showed good predictive power, with the cross-validated R^2^s ranging from 0.48 to 0.73 for individual networks, and 0.50 to 0.75 for network ensembles. Model aggregation improved the results in all cases, both in terms of predictive ability (the R^2^_CV_s of the ensembles were on average 0.02 units greater than those of the corresponding individual predictors) as well as robustness (standard deviation was reduced by a factor of two to three). The training R^2^’s were on average 0.05 units higher than the cross-validated R^2^’s.

**Fig 4:**
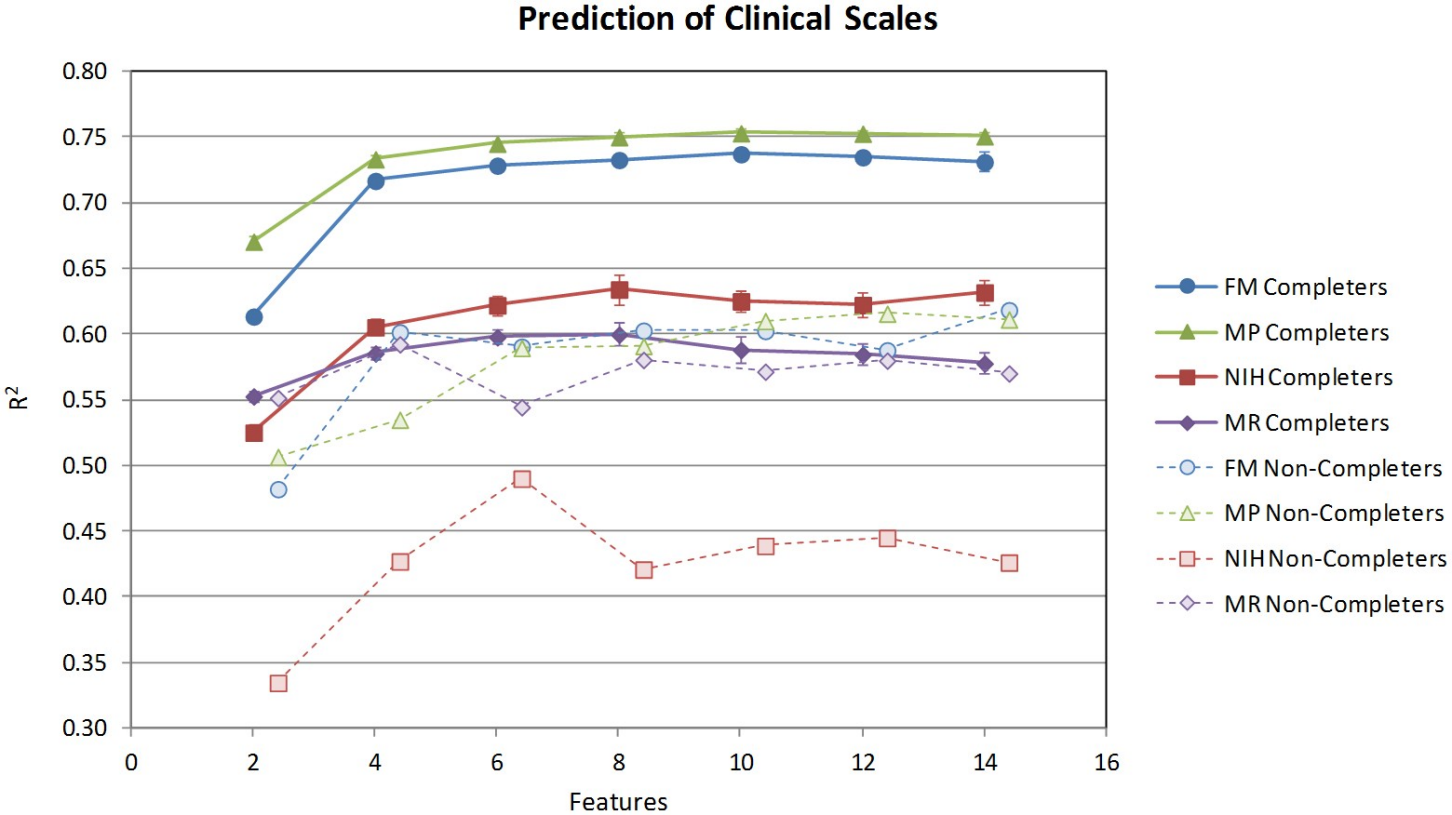
Cross-validated R^2^ of the best models derived from the completers (solid lines) and validated with the non-completers (dashed lines) for each of the four clinical scales, using 2, 4, 6, 8, 10, 12 and 14 robot-derived RMK features. The figure shows the ability of the robot-derived RMK models to predict the clinical scales with an increasing number of features. The model performance exhibits an asymptotic behavior with respect to the number of RMK features, reaching the point of diminishing returns at approximately 8 features for all four clinical scales. Note the small variance in the prediction of the trained data as shown by the small “whiskers,” which for the most part, are not visible in the figure.

The results are summarized in Fig 4. The solid lines represent the average ensemble cross-validated R^2^ of the best model for each clinical scale as a function of the number of input features, aggregated over all cross-validation runs. This plot reveals several trends. First, model performance exhibits an asymptotic behavior with respect to the number of features, reaching the point of diminishing return at approximately 8 features for all four clinical scales. Second, the models for the FM and MP display comparable predictive power, which is not surprising given the strong correlation between them. Further, performance was not uniform across the four clinical scales. The FM and MP models are more predictive than those for NIH and MR, with the latter showing substantial deficits both in terms of predictiveness (mean) and robustness (standard deviation). It should be noted, however, that the MR models were derived from substantially fewer training samples, as we only had data available for days 30 and 90. At the optimal 6–10 feature range, the R^2^_CV_ for NIH and MR are 0.1–0.13 units lower than that of FM and MP, which suggests that the NIH and MR scales encode additional elements of patient function that are not captured by the robotic assay or are inherently more noisy.

More importantly, the ensemble models retain much of their predictive power on the non-completer population, as illustrated by the dotted lines in Fig 4. These are patients who were not ‘seen’ by the model during training, and represent an excellent external validation set. The R^2^ of the best 8-feature models for FM, MP and MR are 0.60, 0.59 and 0.58, respectively, while that of NIH is 0.42. Although these values are lower than those obtained from cross-validation, the models are still highly predictive. Again, for most of the clinical scales, performance plateaus at around 8 features with the exception of NIH where there is a spike in predictive power for 6 features.

Our models are markedly better than those derived by Bosecker et al. on patients with chronic stroke [14]. To reduce the number of dimensions, in that work we used PCA on all kinematic and kinetic metrics as a filter prior to regression, and used multi-linear regression to derive the actual models. The observed differences in predictive power could be due to a number of factors, including the type and number of patients employed in the two studies, the use of neural networks as opposed to multi-linear regression (MLR), and/or the use of a more elaborate feature selection algorithm. To directly compare the two methods, we used the same PCA technique to select the 8 most important features, and derived new multi-linear regression models using our new training set (the same completer population). The 8 features identified by PCA were all on the affected side and included *Aim*, *Deviation*, *Mean Speed*, *Peak Speed*, *Smooth J1*, *Ellipse*, *Rnd Dyn Mean Dist*, and *Mean Z*. In addition, to further distinguish the effects of feature selection and linearity, the 8 features that produced the best neural network ensembles by cross-validated R^2^ for each clinical scale were used to construct equivalent linear models by MLR. All of these models were cross-validated using the same 10-fold cross-validation procedure described in the Methods session, so that their generalization ability could be directly compared.

**Fig 5:**
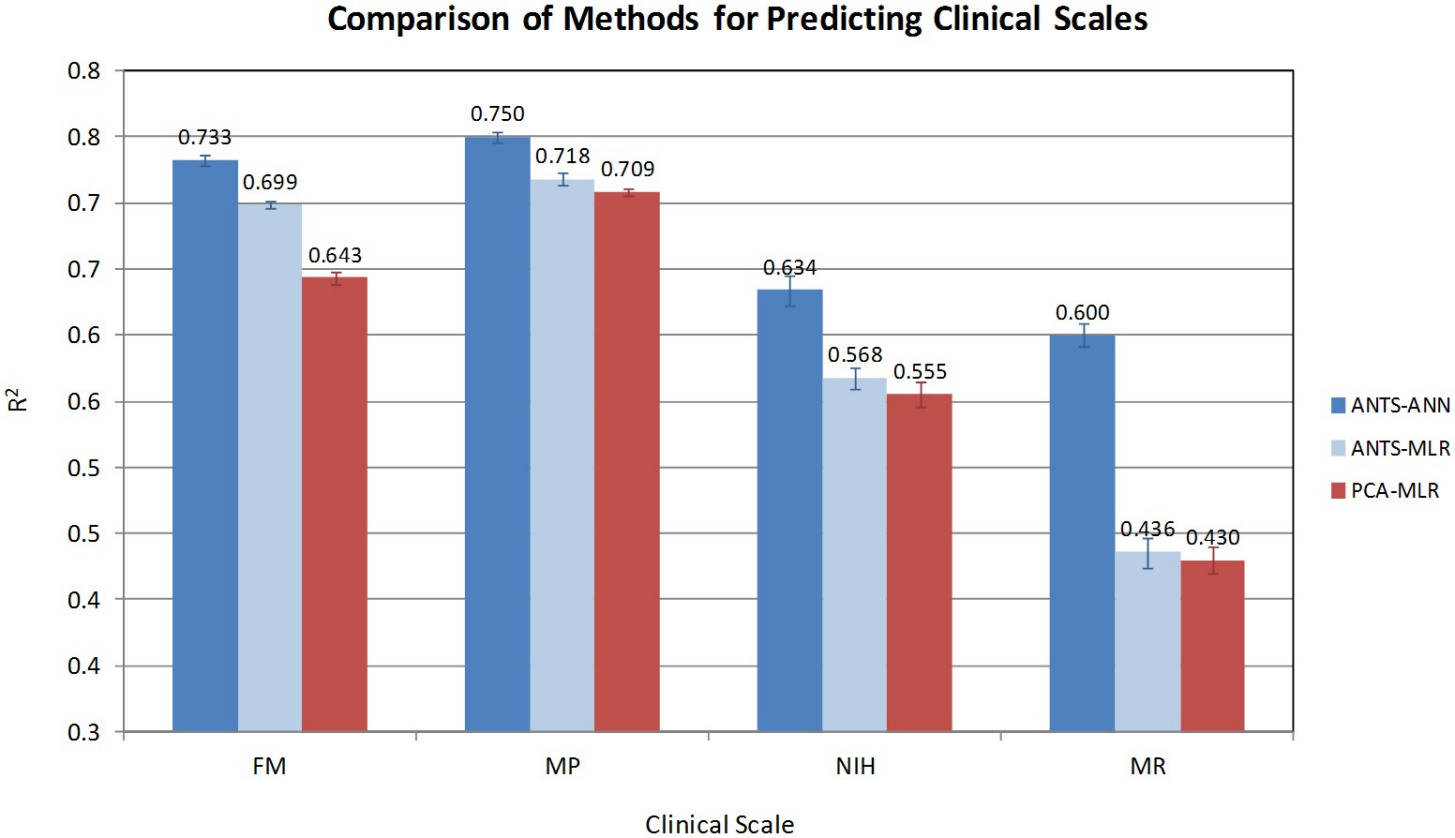
Cross-validated R^2^ of the best 8-feature models derived using three different approaches for feature selection and model building: 1) features selected by artificial ants with neural networks, model derived with neural networks (dark blue); 2) features selected by artificial ants with neural networks, model derived with multi-linear regression (light blue); and 3) features selected by PCA, model derived with multi-linear regression (red). Every model was cross-validated using the same 10-fold cross-validation procedure described in the Methods session.

As can be seen in Fig 5, the use of neural networks coupled with the artificial ant algorithm afford significant benefits, improving the R^2^ by 0.09 for FM, 0.04 for MP, 0.08 for NIH, and a dramatic 0.17 for MR. Most of these gains appear to stem from the use of nonlinear modeling; the difference in the R^2^ between ANTS-MLR and PCA-MLR for FM, MP and MR is only ~0.01, except for NIH where the features selected by the artificial ants are markedly better even in a linear context. These differences are only partly explained by the use of ensembles, which accounts for only a ~0.02 improvement of R^2^ compared to individual networks. (Note that MLR is a stable predictor that does not benefit from this particular form of aggregation.).

### Importance of individual features

One of the goals of our analysis was to gain a more quantitative understanding of what each of the clinical instruments is trying to measure, how they differ from each other, where they fall short, and how we can design alternative scales with greater sensitivity and ability to detect finer differences in motor function.

Given the complex correlation structure of the RMK metrics, the features that are selected by the feature selection algorithm are not necessarily the only ones that can produce a high quality model. The SPE map in Fig 3 shows clusters of highly correlated RMK metrics which could, in theory, be interchangeable in the models. Further, there may be features that can be replaced by a small group of other features as opposed to a single one and still yield a model of comparable predictive power.

**Fig 6:**
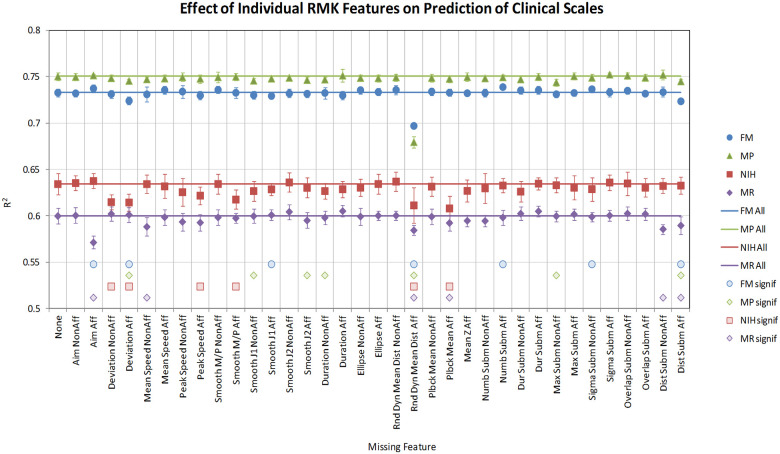
Importance of individual RMK features in predicting the four clinical scales, obtained by systematically removing each feature from the training sample and repeating the feature selection, aggregation and cross-validation procedure for each derived data set. Only models with 8 input and 2 hidden units are shown. The left-most data point and the horizontal solid line on the top part of the plot represent the cross-validated R^2^ of the best model with all features included, averaged over all 10 cross-validation runs (standard deviations shown as error bars). Each subsequent point shows the R^2^ of the corresponding apo model, i.e., the model derived by omitting the feature shown on the x axis (the model still includes 8 features, just not the one shown on the x axis). The individual markers at the bottom part of the plot indicate whether there is a statistically significant difference between the R^2^ distributions of the all-feature and the respective apo models (the presence of a marker indicates that the difference between the two distributions is statistically significant, and the absence that it is not).

To assess the importance of each feature in predicting the various clinical scales, we systematically removed each feature from our training sample and repeated the feature selection, aggregation and cross-validation procedure for each derived data set. Given the computationally intensive nature of this exercise and our previous observations regarding the optimal number of features and hidden units, this process was only tested with models with 8 input and 2 hidden units. The results are summarized in Fig 6.

The left-most data point and the horizontal solid line on the top part of the plot represent the cross-validated R^2^ of the best model with all features included, averaged over all 10 cross-validation runs (standard deviations shown as error bars). Each subsequent point shows the R^2^ of the corresponding *apo* model, i.e., the model derived by omitting the feature shown on the x axis. The individual markers at the bottom part of the plot indicate a statistically significant difference between the R^2^ distributions of the all-feature and the respective *apo* models (the presence of a marker indicates that the difference between the two distributions is statistically significant, and the absence that it is not).

For FM and MP, the most critical feature is *Rnd Dyn Mean Dist Aff*, and its omission results in a 0.04 and 0.07 drop in R^2^ for FM and MP, respectively. *Deviation Aff* and *Dist Subm Aff* also appear important for both scales, but the effect is small (0.01 on the R^2^ scale) and could be an artifact of the small sample size (only 10 cross-validation runs). This confirms that both the FM and MP scales are motor impairment-based scales and can rely on the ability of a patient to exert force in a coordinated manner, which can be captured reliably by a robotic device.

A number of features appear significant for NIH, including *Rnd Dyn Mean Dist Aff*, *Plbck Mean Aff*, *Peak Speed Aff*, *Smooth M/P Aff*, *Deviation Aff* and *Deviation NonAff*. This suggests that the NIH stroke scale has a more global and coarser nature Finally, for the MR scale, *Aim Aff* emerges as the most important feature, followed by *Rnd Dyn Mean Dist Aff*, *Plbck Mean Aff*, *Mean Speed NonAff*, *Dist Subm Aff*, and *Dist Subm NonAff*. The large number of relevant features suggests that the MR scale is coarser than the three other clinical scales, which is not surprising given the low correlation between them (Fig 4).

*Rnd Dyn Mean Dist Aff* is the only feature that is important for all four clinical scales, and its effect eclipses that of any other RMK variable (except *Aim Aff* for MR). This finding, along with the high generalization ability of all the models, suggests that these clinical instruments depends heavily on a person’s motor impairment measurement and as such can be easily replaced with an objective device. It also means that other potentially other relevant aspects of stroke recovery may be missed by these scales, e.g. depression level. While it is not surprising that the features that emerge as most critical in deriving nonlinear models are also among those most correlated to the clinical scales (see Fig 4), it is also clear that the importance of a feature cannot be deduced solely from correlation or in isolation from other feature combinations. Most features can be replaced by other features or groups of features, and many configurations exist that can yield equally predictive models.

### Maximizing effect size with novel composites

**Fig 7:**
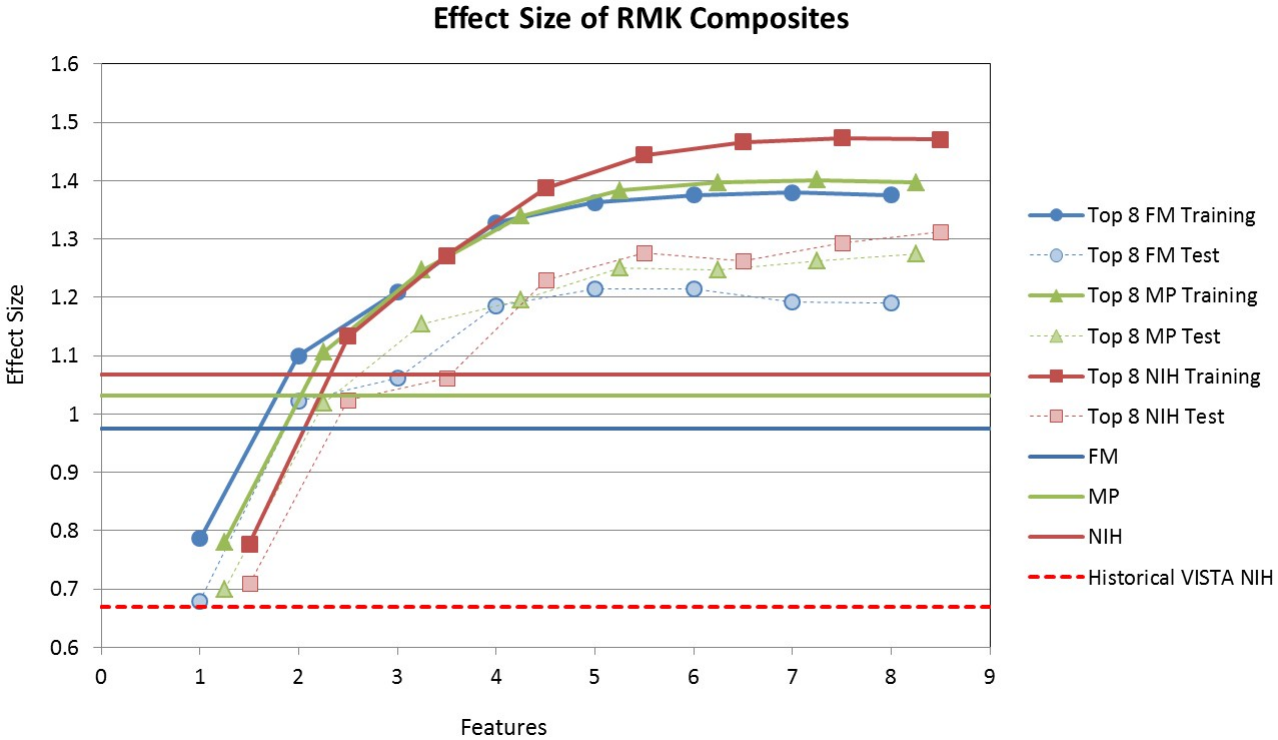
Optimization of effect size for robot-derived RMK metrics. The horizontal lines show the day 7 to day 90 effect size for comparable patients of the historical VISTA data for the NIH, as well as the effect sizes for the NIH, FM and MP assessment scales for our *completers* cohort. The figure also shows the performance of the robot-derived RMK composites optimized for effect size for the trained (solid lines) and cross-validated sets (dashed lines). Note the increase of over 20% in cross-validated effect size for the RMK composites over the clinical scales with 4-features for this study (and over 70% over the historical data).

At this point, we have demonstrated that a small number of RMK invariants can predict the clinical scales with sufficient accuracy to serve as a proxy for measuring impairment in an objective and unbiased manner. As seen in Fig 7, minimizing the inter-rater variability leads to a significant increase in the effect size vs. the historical average. However, if we simply replicated a scale in which there are significant floor or ceiling effects we would not be taking full advantage of the InMotion apparatus. For instance, in this trial, there are individuals with a FM score of 66, which is well within the normal range. These individuals had a stroke that was mild enough, such that it would be difficult to assess improvement over time with the traditional scales.

Thus, our second goal was to determine whether we could improve the sensitivity of the clinical endpoints by means of a novel RMK-based composite that could be used to measure effect size in future clinical trials. We have already seen that motor impairment play a dominant role in all clinical scales, but the relative weight of each of these components differs from one scale to another. We hypothesized that by rebalancing these weights we could detect finer improvements in a patient’s condition over a short period of time. Therefore we sought to create a composite scale made exclusively of RMK metrics and determine whether it could improve our ability to distinguish patient improvement from day 7 to day 90. While no treatment was administered, our assumption was that there would be some natural recovery during the acute and sub-acute stroke phase.

Effect size was assessed using Cohen’s d for paired observations, defined as the mean divided by the standard deviation of the day 7 to day 90 changes over the entire completer population. The composite itself is defined as a linear combination of RMK features:

$$c(j)=\sum_{i=1}^{35}w(i)\cdot rmk2(i,j)$$ (5)

where *RMK*(*i*, *j*) represents the *i*th feature of the *j*th patient, and *w*(*i*) represents the weight of that feature. Eq 5 implicitly assumes that *w*(*i*) will be non-zero for only a specific number of features. To ensure clinical relevance, we set the maximum number of features with non-zero coefficients to 8, and limited the choice to only the features found by the neural networks (we examined separately the three groups of 8 features found in the best models of the three clinical scales). Thus, given a particular set of 8 features, the challenge was to identify the subset of features and corresponding weights that maximized Eq 5. (Unlike the fitting of clinical scales, maximizing the effect size using a nonlinear model would make the problem ill-posed [50]. With prediction, there is a cap on how well a model can perform: a perfect prediction. There is no such intrinsic upper bound on the standard effect size, which can, in theory, be driven to infinity with the use of higher order terms.).

We solved this problem using a greedy forward selection algorithm. Briefly, the algorithm constructed composites by adding one feature at a time until all 8 preselected RMK endpoints were included. The process started by identifying the feature that yielded the maximum effect size and assigning to it a weight of 1. Each remaining feature was then examined in turn, and the one that yielded the largest effect size in combination with the previously selected feature was added to the composite. The algorithm continued in this fashion progressively building larger composites until all 8 features were included. At each step, each candidate feature was evaluated using 18 discrete weights ranging from -1 to +1 in increments of 0.1, while keeping the coefficients of the already selected features at their previously optimized values. Once the feature was selected, the weights of all the features in the current composite were refined in an iterative fashion until the effect size no longer improved. (An alternative backward elimination algorithm was also employed but produced inferior results. That method started by including every preselected RMK endpoint in the composite and optimizing their coefficients using the Newton-Raphson gradient minimization procedure. The feature with the smallest weight was then identified and removed from the composite, the weights of the remaining features were re-optimized, and the process continued in the same fashion until a single feature remained.).

As with the prediction of clinical scales, cross-validation is necessary to ensure that the resulting composites are meaningful beyond the training set. Thus, for each of the three groups of 8 features used in the most predictive models of MP, FM, and NIH, respectively, the forward selection algorithm was repeated 100 times, each time using a different, randomly chosen 80% of the patients to build up the composites and reserving the remaining 20% for testing.

The results are summarized in Fig 7, which plots the average effect size for the training and validation sets as a function of the number of features included in the composite (each point represents the average of 100 cross-validation runs). The blue, green and red solid horizontal lines represent the effect sizes of the FM, MP and NIH clinical scales, and serve as reference points for evaluating the sensitivity of the RMK composites. Overall, the NIH stroke scale is the most effective in assessing change from baseline (effect size of 1.07), followed by Motor Power (1.03) and Fugl-Meyer (0.97).

For the RMK measurements, no single feature performs as well as the clinical scales, which is not surprising given that the latter encapsulate multiple RMK measures, as demonstrated earlier. The effect size increases sharply as additional features are added, exceeds the clinical scales by as much as 30% for the training and 20% for the validation set with only four features, and then plateaus offering little additional improvement. As expected, the effect size is significantly lower for the validation than for the training set and is also more variable.

In order to test the sensitivity of these robot-assisted surrogate markers against historical data, we selected a subset of 2,937 patients from the Virtual International Stroke Trials Archive (VISTA) [51, 52] with a comparable degree of stroke severity as measured by the NIH at day 7. These historical patients were chosen at random so as to match the frequency distribution of NIH scores at day 7 of our current study population, which ranged from 0 to 24. More specifically, for each one of our current study patients, nearly 14 subjects were selected at random from the VISTA registry with the same NIH score measured at day 7. This process yielded a cohort that had the same proportion of patients within each NIH point. This group of 2,937 patients had a day 7 mean NIH score of 5.7 ± 4.1 that improved by 2.1 ± 3.1 points at day 90, giving a standardized effect size of 0.67 for the changes from day 7 to day 90 (illustrated by the red dashed line in Fig 7).

As seen in Fig 7, optimized RMK composites with as few as four features increased the effect size over the historical VISTA level by as much as 107% for the training and 83% for the validation set. This result is highly significant and cannot be attributed to motor learning, as we demonstrated the stability of the RMK measurements on both persons with chronic stroke and age-matched controls. An increase of 83% in effect size compared to the validated set would result in a 70% reduction in the number of patients required to achieve the typical 80% statistical power in a clinical trial. It is worth pointing out that the increase in effect size seen in this natural history trial is not the effect size that one would expect to see in a conventional clinical trial, which is designed to compare the effect of therapy over placebo. It must also be noted that clinical scales represent the best case scenario of relying on a handful of highly trained clinical raters, whereas the composite ought to be highly consistent across multiple sites due to the lack of subjectivity in the assessment.

While the underlying RMK metrics were identical to those used to reconstruct the NIH stroke scale, our modeling effort reweighted each of the individual components, enhancing our ability to detect smaller improvements in our patient population. One surprising aspect of our modeling was that sub-movements do not seem to play a significant role in the new composites. We speculate that sub-movements features might be less important when dealing with coarse aspects of motor abilities and the clinical scales generally employed to measure them.

**Fig 8:**
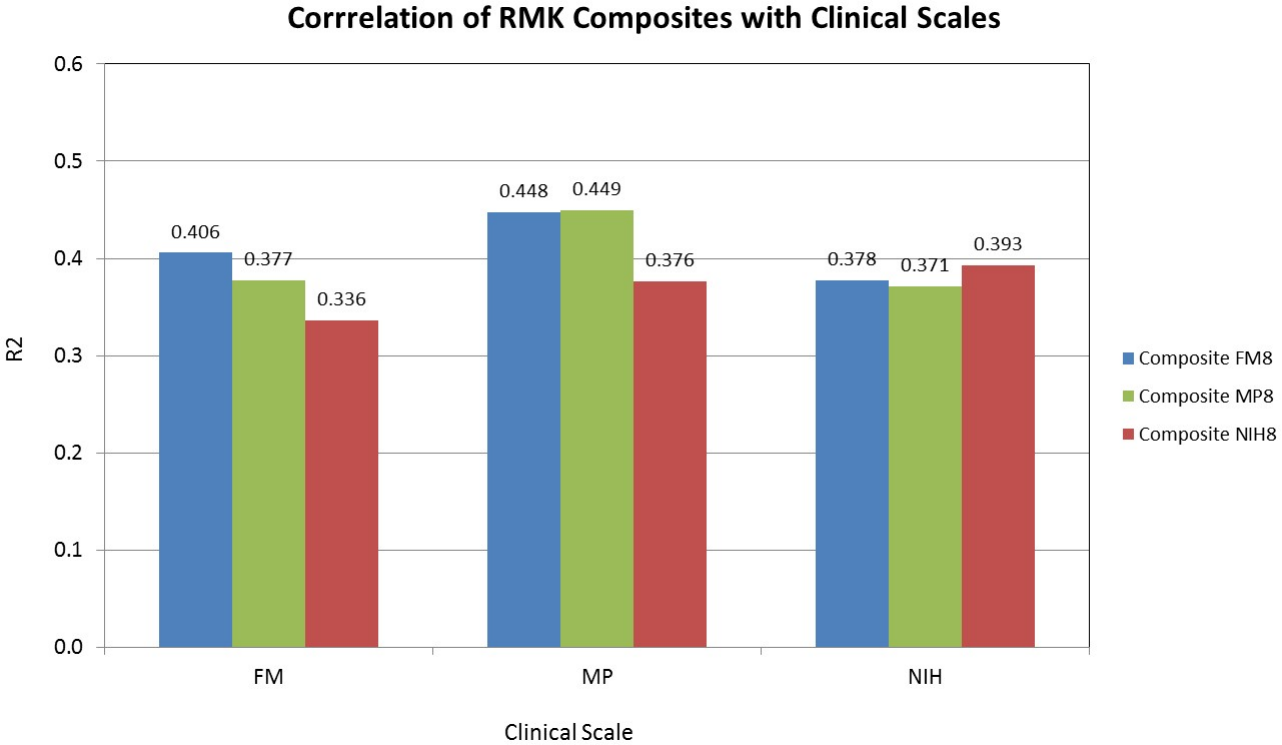
Correlation (expressed as R^2^) of the FM, MP and NIH original scales with the corresponding optimized 8-feature composites obtained with the greedy algorithm.

As illustrated in Fig 8, the composites obtained with the greedy algorithm are correlated with all three clinical scales, with the R^2^s ranging from 0.34 to 0.45. On the basis of these results, we would recommend the composite derived from the 8 features identified by the best NIH neural network model, since it offers the largest effect size for both the training and the validation set, and shows the most consistent correlation with all three clinical scales (0.37, 0.38 and 0.39 for MP, FM, and NIH, respectively).

These results strongly suggest that robotic measurement of motor function may be a viable and improved method for capturing clinical outcomes over traditional clinician-rated measures, and can greatly reduce the sample size required for future clinical trials, thus improving study cost and efficiency. The computational methodology described in this work is not limited only to stroke, but can be applied also to a broad range of problems in medical diagnostics and remote monitoring. While the general clinical findings were described in our earlier publication, the present paper offers greater insights into the relative significance of strength and coordination, and the importance of each robotic feature in capturing different aspects of stroke recovery.

### Future work

In this work, we have established that we are able to accurately replicate the traditional stroke evaluation scales from robotic measurements with a high degree of accuracy, and that a straightforward re-weighting of the features needed to reconstruct the traditional scales can yield a novel composite that is significantly more sensitive than the traditional scales for measuring improvement over time. This has allowed us to establish that, for a fixed time interval, we can greatly reduce the number of patients needed to power a clinical trial.

Our current work has established a composite that works well over the 90 day assessment window. However, given sufficient amounts of data, it would be possible to tune the composite for a patient’s individual level of impairment. The reason for this is that stroke recovery may not progress in a consistent fashion, and initial recovery to stoke may be more sensitive on some metrics vs others. Being able to identify and pre-specify sensitive composites over a specific range of severities would allow us to better tune it towards a patient population and further shrink the number of patients needed for a given clinical trial. Given the design of this trial and the confounding issue of patient improvement over time, it was not possible for us to assess the inherent variability in human performance. Answering this question could provide valuable insights into determining how long a trial needs to run for patients to stand a good chance of showing functional improvement to their physical well-being.

## Conclusions

The results described above are extremely promising but must be interpreted with appropriate caution. The population enrolled in our study was highly selected, with a day 7 mean NIH score of 5.7 ± 4.1. Clearly, any gains in statistical power will need to be balanced against lower enrollment rates imposed by the selection criteria and against potential failure to complete follow-up or to comply with the RMK measurements. Additionally, while the RMK lends itself to repeated assessments to produce averaged measurements, the same might be true if we employ ordinal analysis, central adjudication by multiple raters, and global testing procedures that combine complementary scales across clinical domains and across time (albeit at much greater expense). Finally, there was substantially greater improvement in the NIH scores achieved by our current pool of *completers* compared to historical patients with comparable variability (VISTA). The pool of 2,937 patients selected in the VISTA registry to match the distribution of our pool of 208 patients at day 7 had an improvement of 2.1 ± 3.1 by day 90, while our *completers* had an improvement of 3.7 ± 3.3. This difference must be interpreted with appropriate caution given that the historical patient data was obtained at a different time and in different institutions, a factor that inevitably introduces some bias.

Despite the generally limited penetration of robotic technologies in the post-stroke neurorehabilitation arena (only 200 InMotion Arm robots have been produced so far), taken together, our results suggest that robotic measurements may enable early decision making in clinical testing, reduce required sample sizes, and offer a more reliable method to track longitudinal change in patients affected by stroke than using current clinical instruments. More importantly, this study marks a novel beginning for technology-enabled measurement of outcomes, and offers a proof-of-principle for other robotic and wearable devices potentially affording further improvements and efficiencies.

## Supporting information

S1 Data (CSV)
